# The Influence of Nano CaCO_3_ on Nucleation and Interface of PP Nano Composite: Matrix Processability and Impact Resistance

**DOI:** 10.3390/polym13091389

**Published:** 2021-04-25

**Authors:** Meshal Al-Samhan, Fatema Al-Attar, Jamal Al-Fadhli, Mustafa Al-Shamali

**Affiliations:** Petroleum Research Center, Kuwait Institute for Scientific Research, PO. Box 24885, Safat 13109, Kuwait; fattar@kisr.edu.kw (F.A.-A.); jfadhli@kisr.edu.kw (J.A.-F.); mshamali@kisr.edu.kw (M.A.-S.)

**Keywords:** interface, nucleation, nano CaCO_3_, polypropylene, nanocomposite, NMR, thermomechanical

## Abstract

Polypropylene (PP) is a commodity material that has been increasingly used in different industries in the past two decades due to its versatile properties when enhanced with additives. Homo polypropylene, in general, has weak mechanical properties and limited chemical resistance; thus, using a different type of fillers to adjust such properties to fit the required applications opened a large market for this commodity. Understanding the interface constituent between the polymer matrix and the added filler and the nucleation behavior is a key to fine control of the enhancement of PP properties. In this study, PP was incorporated with nano calcium carbonate (CaCO_3_) at 2 and 5 wt% in the presence of maleic anhydride (MAH) to overcome the weak interface due to low polymer polarity. The mix was compounded in a twin screws extruder at a temperature range of 180–200 °C ; then, the prepared samples were left to dry for 24 h at 25 °C. Nuclear Magnetic Resonance (NMR) was used to study the interface adhesion of the nanofiller and the curved revealed that at 2% of nano CaCO_3_ PP structure remained the same and the nano experienced good adhesion to the polymer matrix. The mechanical impact resistance results showed a real enhancement to the polymer matrix of the nanocomposite by 37%. Moreover, DSC results showed a faster crystallinity rate due to the nanofiller acting as a nucleating agent and rheology tests indicated that low content of nano additive (2%) has better processability behavior, with suitable viscosity complex values at high frequencies.

## 1. Introduction

The advancement in nanomaterials and their applications over the last two decades has opened the research gate widely towards the incorporation of nanoparticles in the polymer matrix to enhance and tailor properties of different polymers. Nanoparticles, whether organic (chitosan) or inorganic substances (hydroxyapatite and calcium carbonate), can be considered as fillers and can be introduced to the polymer in solid or liquid state mixing processes [1,2]. The nanoparticles commonly exhibit many advantages over other fillers (microparticles): higher specific surface area, surface energy and density compared to microparticles [3,4,5]. Interface comportment between the polymer and the nanofiller varies greatly depending on the nature and the surface of the nanoparticle as well as on the polymer matrix [6,7]. The reinforcement of NPs, matrix and the interfacial region are the main constituents in any composite, where the latter is responsible for properties dissimilar from the major matrix due to its proximity to the filler surface [8]. The factors that hamper the reinforcement of nanostructures are alignment, dispersion of the filler and interfacial bonding between the filler and polymer [9]. Many studies reported that better mechanical properties are attributed to the formation of an interfacial adhesion layer in the presence of a bonding agent such as cationic surfactants or maleic anhydride (MAH) compatibilizer [10,11]. The simultaneous introduction of both filler and compatibilizer is beneficial, as the compatibilizers are frequently used to improve the interfacial adhesion between fillers and the matrix result in optimum dispersion which is usually difficult to achieve [12,13]. In a previous work [14], we showed that nano calcium carbonate CaCO_3_ exfoliation contributed to better elastic behavior of the nanocomposites indicating that the physical cross-link toughens the nanocomposite and give higher storage modulus above the glass transition temperature (Tg). 

For a semicrystalline PP composite, the interactions at the interface depend on reactive groups of the filler at the surface, matrix morphology and filler shape. The interface influences the composites thermomechanical properties; Lopattananon et al. [15] reported that the properties of a thin interface impact the nature of load transfer, displaying full debonding, while others did not. Therefore, understanding the nanofiller attachment to the interface is a significant factor in optimizing the mechanical property of the nanocomposite. A weak interface decreases the efficiency of stress transfer from the matrix to the nanofiller and consequently reduces strength and stiffness [16].

On the other hand, nucleation of the semicrystalline polymer due to the introduction of the nanofiller is another important phenomenon that contributes to the properties of the nanocomposite and small particle sizes generally make for more effective nucleators. When the nanofiller acts as a nucleation agent and increase the crystallization rate, eventually, this will lead to speed crystal formation and changes in spherulite size [17,18]. This cycle of crystallization of the nanocomposite affects the density of the polymer and create a room for properties improvement. Huajie Mao et al. [19] reported that nano-silica and nano-CaCO_3_ are used as a nucleating agent for PP that affect cell structure. In addition, Ding et al. reported that the low contents (5%>) of nano-CaCO_3_ exhibits an optimum cell structure [20,21].

This study covers an extensive knowledge of the behavior of the interface and its impact on mechanical and processability properties of PP incorporated with nano CaCO_3_ in the presence of MAH. The rheological analysis was carried out to provide information about complex viscosity, storage and loss moduli data, which are useful to adjust the processing conditions. It is essential to evaluate the processability of the nanocomposites for production as well as for recyclability.

## 2. Materials

PP is available commercially in various grades. In this study, homo PP was acquired from a local vender, Kuwait. The high purity NCaCO_3_ (In fine powder form, Whiteness (%)>90, Bulk Density (g/mL) 0.68) with average particle size of 15–50 nm purchased from American logistics company through a local agent, where CaCO_3_ (In powder form,) and MAH (in crystal form) were all acquired from international manufacturers through KISR’s store department.

## 3. Sample Preparation and Testing

Different batches of compositions were prepared: polypropylene alone (as the base matrix) and PP with nano-CaCO_3_ 2 and 5 wt%. The batches also mixed with varying surface compatibilizers load (2 and 4 wt%). Each batch was thoroughly mixed for 120 min dry-mixed with polypropylene (PP) to ensure adequate dispersion of the filler particles. The different mixes were subjected to compounding using a twin-screw extruder (model latch LTE 26/40) at 200 °C and the materials were fed by a vertical hopper with 2 kg/h feeding rate then the final samples left to dry at room temperature for 24 h. All prepared samples were cut in standard shapes according to each test requirement.

To assess the impact resistance, the prepared samples were tested using INSTRON Ceast 9050 at ambient temperature with the impact angle fixed at 160 degrees.

To assess the structure, the Nuclear Magnetic Resonance (NMR) 400 MHz was used under solution technique; the nanocomposite material was ground then mixed with Acetone d^6^. Proton one degree NMR pulse sequence was applied for each sample (pure: 2% and 5%) separately (a 90-degree pulse sequence; proton is the name of the pulse in Topspin; one degree pulse sequence; duration time, 26 usec; relaxation time, 2 sec; power, 18.911 w; number of scans, 100 scans). The NMR spectra were obtained for both samples and analyzed using BRUKER software. The polymer partially dissolved in acetone so that it can be detected by NMR.

To assess the DSC (differential scanning calorimeter), DSC measurements were carried out using a DSC-60 manufactured by Shimadzu, Japan. The starting temperature was 25 °C and then the temperature was increased gradually to 300 °C. The temp rate was 10 °C/min in cooling and heating directions.

To assess the rheology, a rheometer manufactured by TA Instruments Model HR 3 Discovery (Germany) used to evaluate the nanocomposite processability, the angular frequency dependence of shear storage modulus (*G*′) and shear loss modulus (*G*″) with complex viscosity (η*) at set conditions (temperature: 180 °C; geometry: 25 mm parallel plates; procedure: frequency sweep; gap: 1000 mm, angular frequency: 0.1 to 200 rad/s).

In this study, experiments and other tests were performed three times and the arithmetic mean values are reported.

## 4. Results and Discussion

### 4.1. Interface Evaluation 

#### 4.1.1. Mechanical Comportment: Impact Resistance

Impact resistance to evaluating the mechanical performance of the PP nanocomposites the of the prepared samples are reported in this part. In a straight explanation, a polymer reinforced composite is composed of three constituents: the filler, the matrix and an interface responsible for assuring the bond between the matrix and filler [22]. 

**Figure 1:**
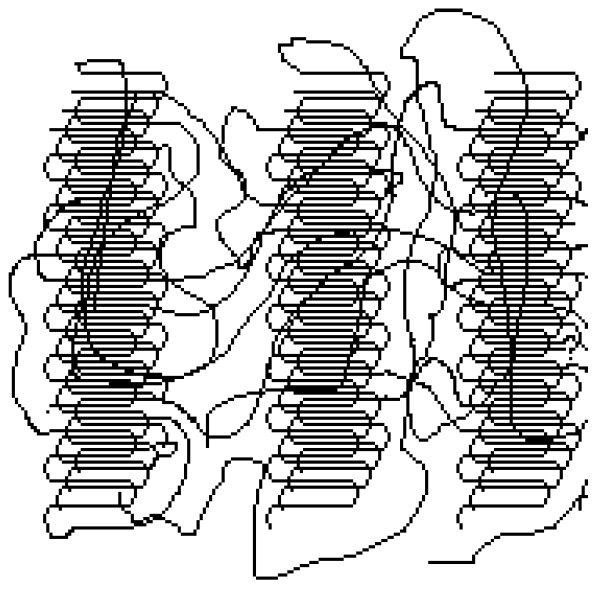
Lamellae crystalline portion with the amorphous portion.

The impact evaluation of nano-CaCO_3_ presented in Table 1, for homo PP the impact resistance as a semicrystalline material is low to average close to that of amorphous plastics. The presented results for the impact resistance show a noticeable improvement for all nanocomposite samples in comparison with the pure PP results. Moreover, reaching an increase of 10% when using a 2 wt% nanofiller load This valuable increase in the impact resistance indicates that the nanofiller has strengthened the bonding between lamellae crystalline portion with the amorphous part outside the lamellae, as shown in Figure 1 [23]. However, for the 11%, it is noticed that the more increase in nano content does not improve the impact resistance further. Perkins reported that the size of the filler particle is critical and the large particle size act as weaknesses and susceptible to cracks [24].

Moreover, distinct from fiber filler reinforcement resistivity to impact which depend on the fiber direction (perpendicular) to the applied force, the nano-CaCO_3_ has no direction within the matrix or at the matrix interface, which results in the reinforcement of the polymer nanocomposite equally in all direction to the applied force.

Over-all the impact resistance test demonstrated that the introduction of nanofiller dramatically enhanced the strength of PP composite samples confirming that size of filler particles can reverse the bonding with the amorphous side for the semicrystalline polymer (PP). However, the value of this improvement is of great importance when it synergizes with the other nanocomposite properties such as thermal and chemical.

#### 4.1.2. NMR: Solution Analysis

**Figure 2:**
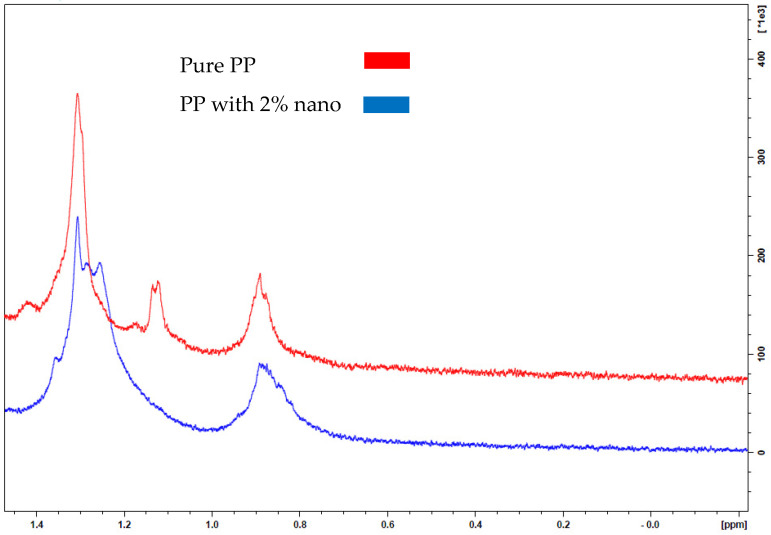
NMR analysis for PP pure and PP nanocomposites with nano-CaCO_3_ 2% wt.

**Figure 3:**
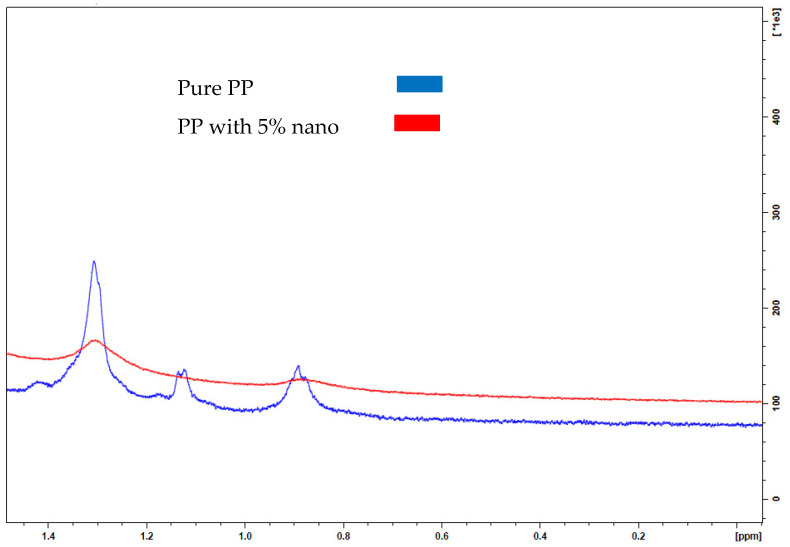
NMR analysis for PP nanocomposites with nano-CaCO_3_ 2% and 5% wt.

The NMR analysis for the prepared nanocomposite samples was carried out according to ASTM standard; the results are shown in Figure 2 and Figure 3. From the graphs with the peaks assigned in Figure 2 and Figure 3, it is clear the sample of 2% NCaCO_3_ has more peaks chemical shift than 5% NCaCO_3_. The chemical shift is most often used for structure determination, through the shield patterns [25]. The 2% NCaCO_3_ and the 5% share some chemical shift related to the pure PP, where 2% NCaCO_3_ sample has more chemical shield than 5% NCaCO_3_. In addition, the chemical shield in 5% is less intense than that for the 2% sample. Correspondingly, as an indication of structural change in the original sequence of the pure PP, the chemical structure the 2% NCaCO_3_ has minor chemical shield difference than the original PP. 

However, 5% spectrum shows some change in the structure when compared with virgin polypropylene, indicating that the increase in nano additive material leads to more interaction with polypropylene and affecting the structure sequence. This illustrates that composite with 2% spectrum shows similar information to the pure PP indicating strong bonding at the interface without restructuring of the lamella. This finding synergizes with the results from the impact resistance inductive of significant interface bonding, whereas the spectrum of the composite with 5% nano has a different peak sequence signifying structural changes of PP. Both spectra show polypropylene sequence with the addition of the chemical molecule. 

This finding can support that nano-CaCO_3_ increase nucleate sites toward higher crystallinity state. Moreover, the NMR analysis implies that there is an effect on the polymer matrix leading to composite properties altering, whether in a positive direction or negative, which can be verified through thermomechanical properties tests. 

### 4.2. Nuclei Behavior

#### 4.2.1. DSC: Crystallinity

**Figure 4:**
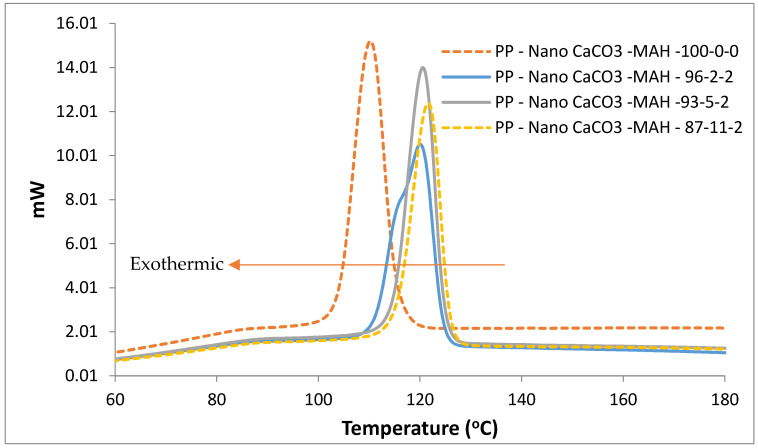
Cooling DSC crystallization curves for PP with different nano-CaCO_3_ wt%.

Nucleating agents such as organic salts, nanofiller particles and ionomers can affect the crystallization; they act as seeds and can increase the crystallization rate. When nano-CaCO_3_ act as nucleation agent to polypropylene, it shows a noticeable increase in the crystallinity; however, once agglomeration of the nanofiller occurs due to the high level of filler content, the nucleating sites lessen and consequently crystallization decreases. The DSC results for PP samples filled with nano-CaCO_3_ at different filler content are shown in Figure 4. The shift in the crystallization temperature indicates that nano-CaCO_3_ acted as nucleation promoters. In addition, Figure 4 shows the increased range of crystallization temperature (Tc) in the crystallization course decreases with the increase of nanofiller content. Therefore, the addition of 2% can give equal nucleation activity to higher filler content which reduces production cycle and consequently save the processing cost. Moreover, interfacial compatibility in the presence of MAH demonstrated good adhesion and allowed an approximate of 11% increase in Tc. A comparison among the data on melting temperature and melting characteristics shows that the compatibilizer concentration has a negligible influence on Tm, as shown in Table 2.

In general, PP nanocomposite results from DSC and NMR demonstrated the addition of a nucleating agent to an unfilled semicrystalline polymer brings about the change of polymer crystallization rate and crystalline structure [26]. However, for the melting point of the nanocomposite, it remained almost the same for all different nanofiller concentrations and MAH contents (Table 2). This could be attributed to stability in the polymer lamellar thickness, which consequently results in thermal stability of the PP nanocomposite stability.

However, if there are many nucleation sites along the surface, then the resulting spherulite growth is restricted in the lateral direction.

#### 4.2.2. Rheology: Processability 

**Figure 5:**
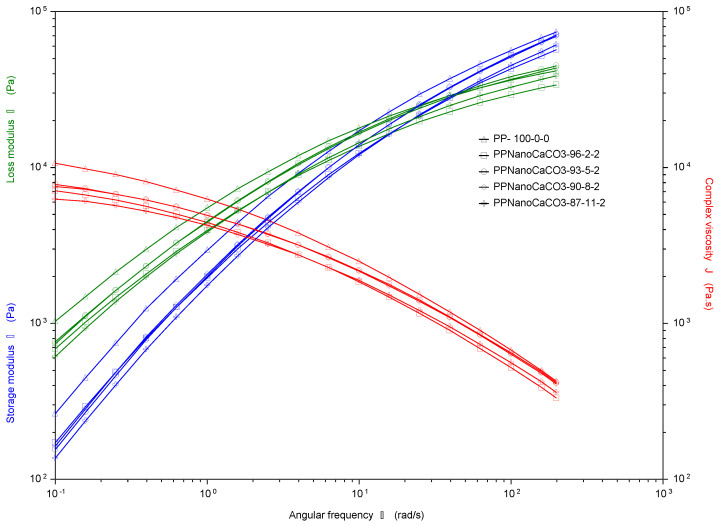
Complex viscosity and storage modulus for PP with nano filling at 2% MAH.

**Figure 6:**
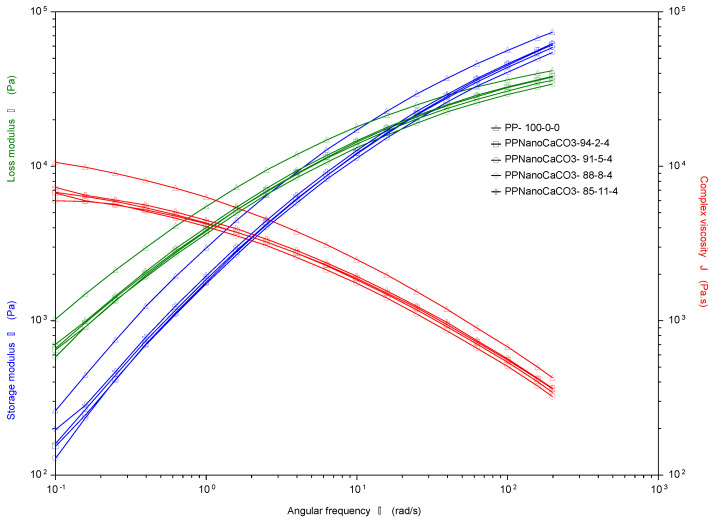
Complex viscosity and storage modulus for PP with nano filling at 4% MAH.

The incorporation of nanofillers in a molten polymer brings a change in the viscoelastic properties, the angular frequency dependence values of shear storage modulus (*G*′) and shear loss modulus (*G*″) with complex viscosity (*η*∗) at the selected conditions are plotted in Figure 5 and Figure 6 for different compatibilizer (MAH) concentrations. The nanofiller at different concentrations had influenced the viscoelastic property of PP nanocomposites, as all prepared nano samples showed less storage modulus and complex viscosity than the blank PP. This is indicate that less energy required for dissipation, considering that elastic behavior of the material under a shorter process of time has a negative effect on its processability and is linked to creep and stress relaxation. Moreover, adding nano CaCO_3_ at different MAH wt % showed similar trend at lower frequency region; however, the modulus of nanocomposite samples was lower compared to pure PP, as mentioned earlier. 

Correspondingly, it is worth noting that at high frequency, the nanocomposite behaved more as viscoelastic earlier for nano concentration 2 and 5%, for the samples with MAH content of 2%. Conversely, for the samples with high MAH concentration (4%), the values of G′ were very tight for all nano CaCO_3_ concentrations, indicating that MAH governs the viscoelastic behavior of the nanocomposite making the concentrations of the nanofiller of a less effect. Thus, for cost-effectiveness, PP with a low volume of nanofiller has the same processability behavior as for the high mixing volume of the nano CaCO_3_. The shear-thinning phenomenon states that polymer melts are non-Newtonian fluids and their viscosity decreases with an increase in shear rate. Accordingly, this behavior is considered the most crucial property in polymer processing. In general, samples with low MAH concentration showed better processability behavior with a suitable viscosity complex values at high frequencies and more responsive to the nano content concentration.

## 5. Conclusions

The study showed that nanofiller could act as nucleating agent and the concentration of the nano-CaCO_3_ greatly affected the crystallization rate, crystalline structure and the degree of crystallinity of the semicrystalline polypropylene nanocomposites and consequently modifying the performance of the material. It was also found that the nanofiller particles size impacted the interface of the polymer matrix can reverse the bonding with the amorphous side for the semicrystalline polymer (PP). This finding revealed an enormous potential to be considered in upgrading homo PP by incorporating of nano-CaCO_3_, mainly for the purposes that need good chemical resistance characteristics, strength, high stiffness and excellence toughness properties and suitable physical property.

## Figures and Tables

**Table 1:** Impact Results for the nano-CaCO_3_ Samples with 4 wt% MAH.

Concentration (wt%)			Impact Energy (J)	Impact Strength (J/m)
PP	CaCO_3_	MAH		
**100**	0	0	2.483	248.3
**94**	2	4	2.697	269.7
**91**	5	4	2.614	261.4
**85**	11	4	2.542	254.2

**Table 2:** DSC melting temperature of PP nanocomposite for different nano-CaCO_3_ and MAH wt%.

Concentration (wt%)			Melting Temp (°C)
PP	NCaCO_3_	MAH	
100	0	0	167.22
96	2	2	166.80
93	5	2	166.38
90	8	2	167.50
87	11	2	167.39
100	0	0	167.22
94	2	4	166.77
91	5	4	166.63
88	8	4	167.68
85	11	4	165.18

## Data Availability

The data presented in this study are available on request from the corresponding author.
